# Effect of Caffeic Acid and Natamycin on the Properties of Poly(butylene succinate) for Packaging Applications

**DOI:** 10.3390/polym18060749

**Published:** 2026-03-19

**Authors:** Lauren Szymańska, Aneta Raszkowska-Kaczor, Oksana Krasinska, Magdalena Stepczyńska, Krzysztof Moraczewski

**Affiliations:** 1 Łukasiewicz Research Network, Institute of Polymer Materials, M. Skłodowska-Curie 55 Str., 87-100 Torun, Poland; lauren.szymanska@impib.lukasiewicz.gov.pl (L.S.); aneta.kaczor@impib.lukasiewicz.gov.pl (A.R.-K.); oksana.krasinska@impib.lukasiewicz.gov.pl (O.K.); 2 Faculty of Materials Engineering, Kazimierz Wielki University, Chodkiewicza 30 Str., 85-064 Bydgoszcz, Poland; m.stepczynska@ukw.edu.pl

**Keywords:** poly(butylene succinate), caffeic acid, natamycin, biodegradable packaging

## Abstract

This study analyzes the effect of two bioactive additives—caffeic acid and natamycin (Natamax^®^)—on the properties of poly(butylene succinate) (PBS) in the context of applications in biodegradable active packaging. Materials containing 1, 3, and 5 wt.% of the additives were prepared by melt blending and characterized in terms of density, rheological behavior (MFR), mechanical properties, thermal stability (TGA), and thermal behavior and crystallization (DSC). Caffeic acid strongly reduced the melt viscosity (reflected by a significant increase in MFR) and, at higher concentrations, led to material stiffening and increased strength at the expense of a pronounced reduction in deformability. Natamycin exhibited a milder rheological effect; at 1 wt.% it simultaneously improved strength and elastic modulus, whereas at higher loadings it deteriorated mechanical performance due to structural effects. Both additives were thermally compatible with PBS; caffeic acid introduced an additional degradation step, while Natamax^®^ did not significantly alter the degradation mechanism. The results indicate that both the type and concentration of the additive govern the structure–property–function relationships and enable the design of PBS-based packaging materials with controlled performance and functional characteristics.

## 1. Introduction

Over the last decade, the packaging sector has become one of the main drivers of the development of biobased and biodegradable materials, driven simultaneously by environmental pressures, consumer expectations, and the intensification of efforts toward a circular economy [1,2]. From a polymer science perspective, a key challenge remains the transition from “nominal biodegradability” to genuinely functional packaging systems that combine: (i) adequate mechanical strength, (ii) barrier properties against oxygen and water vapor, (iii) processing and service stability, and (iv) active functionality, i.e., the ability to limit oxidative processes and microbial growth in order to extend food shelf life [3,4,5].

Among biodegradable aliphatic polyesters, poly(butylene succinate) (PBS) occupies a prominent position due to its good processability in conventional technologies (film extrusion, thermoforming, injection molding), balanced mechanical properties, and the possibility of producing its monomers from renewable resources (succinic acid and 1,4-butanediol). Comprehensive reviews indicate that PBS is one of the most promising “packaging polyesters”; however, its broader application is limited by, among other factors, moderate barrier properties, susceptibility to thermo-oxidative aging under certain conditions, and the need for further improvement of its functionality while maintaining biodegradability [6,7,8]. At the same time, the field of PBS-based coatings and hybrid structures (e.g., paper coating with aqueous dispersions) is developing rapidly, in response to the growing importance of cellulosic packaging with enhanced barrier performance, while maintaining compatibility with recycling and/or biodegradation in appropriate waste streams [9].

An important trend in the design of next-generation packaging is the transition from passive films to active packaging, in which compounds with antioxidant and/or antimicrobial activity are incorporated into the polymer matrix (or functional layer). Reviews devoted to biodegradable active materials emphasize that the effectiveness of such systems does not depend solely on the “chemical activity” of the additive, but on the entire system, including its compatibility with the matrix, dispersion state, thermal stability during processing, migration/release kinetics, and interactions with food and the storage environment [10,11]. In the case of PBS, active packaging systems are being intensively investigated, among others, through the incorporation of nanofillers and sorbents (e.g., carriers capable of ethylene scavenging combined with antimicrobial activity), as demonstrated in studies encompassing both material characterization and application tests on fruits [12,13,14]. The literature also reports PBS-based active films with antifungal activity for fruit packaging applications (e.g., systems containing nanoscale lignin and volatile compounds), in which both an improvement in selected barrier properties and a preservative effect under storage conditions were demonstrated [15].

In this context, natural phenolic compounds, including phenolic acids, are of particular interest due to their multifunctional activity, encompassing free radical scavenging, metal ion chelation, and interactions with microbial cell membranes. Phenolic acids may serve as a versatile platform for the design of biodegradable active materials; however, their use also highlights the dependence of effectiveness on the polymer matrix architecture, system polarity, and transport mechanisms within the material [16,17,18,19,20]. At the application level, for example, the possibility of producing hydrophilic films with controlled antioxidant activity and a described release kinetics of caffeic acid in film-forming systems has been demonstrated, providing an important reference point for the design of functional stability during food storage [21,22,23]. Moreover in [24] the strategies for integrating caffeic acid with biopolymers (physical blending, immobilization, crosslinking, and nanocomposite approaches) were systematized, emphasizing that maintaining activity while limiting uncontrolled migration remains one of the key translational challenges for active packaging.

Beyond caffeic acid, a broad range of other phenolic acids such as ferulic, p-coumaric, gallic, and tannic acids have been investigated as functional additives in polyester-based polymer composites, particularly in systems based on polylactide (PLA), poly(butylene succinate) (PBS), and related biodegradable polyesters [25,26,27]. These low-molecular-weight phenolics are primarily introduced to impart antioxidant and antimicrobial functionality; however, numerous studies demonstrate that they also significantly influence the thermal, mechanical, and structural behavior of polyester matrices. The presence of multiple hydroxyl and carboxyl groups promotes hydrogen bonding and polar interactions with ester functionalities, often leading to restricted chain mobility, changes in glass transition temperature, and modifications of crystallization behavior. Depending on concentration and dispersion, phenolic acids may act either as mild plasticizers or as rigidifying agents, while at higher loadings aggregation effects and reduced interfacial compatibility can adversely affect mechanical performance. Such structure–property trade-offs have been repeatedly reported for PLA and PBS composites containing ferulic, gallic, and tannic acids, highlighting that the effectiveness of phenolic acids in active polyester materials is governed not only by their intrinsic bioactivity but also by their physicochemical interactions with the polymer matrix and processing-induced morphology. These findings underline the relevance of systematically studying different phenolic acids in biodegradable polyesters to balance functional activity with material performance.

Another class of additives of high practical relevance comprises preservatives with documented antifungal activity, among which natamycin (commercially available, e.g., as Natamax^®^) is one of the most well-established solutions in food technology. Review articles devoted to natamycin highlight its effectiveness in inhibiting the growth of yeasts and molds, while simultaneously pointing to the need for an appropriate “polymeric carrier” that ensures stability during processing and controlled release [28]. The literature has demonstrated the effectiveness of natamycin in packaging films and coatings of various natures, ranging from edible chitosan films for cheese (with simultaneous determination of diffusion parameters), through hybrid sol–gel coatings on PLA films, to advanced biodegradable multicomponent systems (e.g., PBAT/PLA with compatibilizers and fillers), in which high efficacy in inhibiting pathogen growth and improved shelf life of stored fruits were shown [29,30,31]. It is worth noting that, despite the growing number of publications on natamycin in biodegradable packaging, a substantial proportion of studies focus on hydrophilic matrices or on polyesters other than PBS, while comparable and systematic data for PBS—particularly in concentration regimes relevant to industrial practice—remain relatively limited. At the same time, studies directly addressing natamycin–polymer systems for packaging applications are already emerging, which confirms the topicality of this subject [32,33].

Despite the growing body of literature devoted to phenolic compounds and antifungal agents in biodegradable polymer systems, systematic studies addressing their simultaneous impact on processing behavior, thermal transitions, crystallization phenomena, and mechanical performance of poly(butylene succinate) remain scarce. In particular, comparative analyses that consider both phenolic acids and non-phenolic bioactive preservatives within the same polyester matrix and under identical processing conditions are largely missing. From the perspective of the current state of the art, three research gaps are particularly relevant and justify the present work. First, in the case of PBS there is still a lack of a coherent description of the effects of selected natural additives on the “property chain” relevant to processing and use, including melt rheology (MFR), thermal stability (TGA), crystallization behavior (DSC), mechanical properties (tensile testing), and structural parameters related to density and packing. Second, phenolic additives such as caffeic acid may act both as antioxidants and as modifiers of interfacial interactions (via hydrogen bonding and polar interactions), which, depending on their dispersion state and concentration, may result in either improvement or deterioration of barrier and mechanical performance; predicting the direction of these changes without experimental data for a given matrix and processing conditions remains highly uncertain [24,25]. Third, natamycin, as an active compound with strong antifungal activity, simultaneously poses material challenges (compatibility, thermal stability, potential agglomeration) and functional challenges (release kinetics, durability of the effect), which require evaluation in the context of the target polyester and at realistic additive loadings [28,33].

In this article, the design and characterization of PBS materials modified with 1, 3, and 5 wt.% of caffeic acid or Natamax^®^ as model “bio-active” additives within the strategy of developing biodegradable packaging is addressed. The selection of PBS is justified by its functional performance and industrial maturity, whereas the choice of additives reflects two complementary functions: (i) stabilization and protection against oxidation (caffeic acid as a phenolic antioxidant with documented activity and compatibility with biopolymers), and (ii) inhibition of mold and yeast growth (natamycin as an antifungal compound with established use in packaging systems). The adopted concentration range (1–5 wt.%) corresponds to the range in which both pronounced functional activity and significant changes in material properties (depending on dispersion mechanisms and interactions with the matrix) have been reported in the literature, making it particularly suitable for defining a “design window” for packaging applications.

The expected contribution of this work is to rationalize the structure–property–function relationships in PBS/caffeic acid and PBS/Natamax^®^ systems, with particular emphasis on parameters critical for packaging implementation, including thermal and processing stability, changes in crystallinity, effects on melt flow, and the mechanical consequences for films and thin-walled products. Such an analysis is of practical relevance for the development of biodegradable materials, as it enables the rational selection of the additive and its concentration in order to achieve the desired functional effect without compromising processability and end-use properties, which is a prerequisite for the transition from laboratory studies to industrial solutions.

## 2. Materials and Methods

### 2.1. Materials

Polybutylene succinate (PBS) polymer granulate (BioPBS FD92PM) was purchased from Mitsubishi Group Chemicals (Düsseldorf, Germany). Caffeic acid (C9H8O4) with molecular weigth of 180.16 was supplied by Glentham Life Sciences (Planegg, Germany). Natamax^®^ containing min. 50% of the active agent (natamycin) and carrier (lactose) was supplied by DuPont Danisco (Marlborough, UK).

### 2.2. Preparation of Composites

The composites were prepared using a Plasti-Corder PLV 151 internal mixer (Brabender, Germany). Caffeic acid (CA) or Natamax^®^ (N) was added to neat PBS at loadings of 1, 3, or 5 wt.%. The materials were processed at 120 °C for 10 min with a rotor speed of 50 rpm. Once the mixtures were obtained, they were pressed on a hydraulic laboratory press into 1 mm thick sheets. Samples were then cut from the sheets for testing. The designation of the samples and the composition of the produced materials are presented in Table 1.

### 2.3. Methodology

The density of the obtained materials was determined in accordance with PN-EN ISO 1183-3:2003], “Plastics—Methods for determining the density of non-cellular plastics—Part 3: Gas pycnometer method” [34], using a ULTRAPYC 5000 Foam gas pycnometer (Anton Paar, Graz, Austria). Prior to the measurements, the samples were conditioned at 23 °C and 50% relative humidity.

The melt mass-flow rate (MFR) was determined in accordance with PN-EN ISO 1133:2022, “Plastics—Determination of the melt mass-flow rate (MFR) and melt volume-flow rate (MVR) of thermoplastics” [35], using a capillary plastometer LMI 4003 (Dynisco, Heilbronn, Germany). The measurements were performed at 190 °C under a piston load of 2.16 kg.

Static tensile tests of the materials containing caffeic acid and natamycin were performed in accordance with PN-EN ISO 527-1:2020, Plastics—Determination of tensile properties Part 1: General principles [36], using a universal testing machine (Instron model 3367, Instron, Norwood, MA, USA). The crosshead speed was set to 50 mm/min. The tensile strength (σM), stress at break (σB), strain at maximum stress (εM), strain at break (εB), and Young’s modulus (E) were determined.

Thermal stability was investigated using a Q500 thermogravimetric analyzer (TA Instruments, New Castle, DE, USA). The measurements were carried out under a nitrogen atmosphere in the temperature range from approximately 25 to 700 °C, with a heating rate of 20 °C/min. The sample mass ranged from 16.0 to 17.0 mg. From the obtained thermogravimetric curves, the temperatures corresponding to 5%, 50%, and 95% mass loss (T_5%_, T_50%_, and T_95%_, respectively), as well as the residue after thermal degradation (R), were determined. The T_5%_ value was used as an indicator of the thermal resistance of the material. The degradation process was further analyzed by determining: the number of degradation steps, the mass loss in individual steps, and the temperatures of the maxima of the respective degradation stages.

Differential scanning calorimetry (DSC) measurements were performed under a nitrogen atmosphere using a Q200 scanning calorimeter (TA Instruments, USA). Samples with a mass of approximately 10 mg were analyzed in the temperature range from −60 to 200 °C at a heating/cooling rate of 10 °C/min. From the cooling curves and the second heating scans, the crystallization temperature (T_c_), crystallization enthalpy (ΔH_c_), glass transition temperature (T_g_), cold crystallization temperature (T_cc_), enthalpy change cold crystallization (ΔH_cc_), melting temperature (T_m_), melting enthalpy (ΔHm), and the degree of crystallinity (X_c_) were determined. The values of X_c_ were calculated according to Equation (1):

(1) $$X_{c}=\left(\frac{{∆H}_{m}}{{∆H}_{m100\%}\times \left(1-x\right)}\right)\times 100\%$$

where ΔH_m100%_—the enthalpy change of 100% crystalline PBS; 110.3 J/g [37].

x—the filler content.

## 3. Results and Discussion

The density of neat PBS was 1.2087 g/cm^3^, whereas the incorporation of small amounts of caffeic acid or Natamax^®^ into the matrix resulted in differentiated changes in this parameter. The density values of the prepared materials are summarized in Table 2.

The addition of caffeic acid at concentrations of 1–5 wt.% caused a slight decrease in the density of the composites (1.2019–1.2054 g/cm^3^), with the observed differences being small and within the range of 0.3–0.6% relative to the reference material. This suggests good compatibility of caffeic acid with PBS and the absence of pronounced disturbances in polymer chain packing. The minor reduction in density may result both from the lower intrinsic density of the additive and from a mild plasticizing effect leading to a slight loosening of the PBS structure.

A different effect was observed for the composites modified with Natamax^®^. Already at 1 wt.% of the additive, the density increased to 1.2217 g/cm^3^, and at 3 and 5 wt.% it remained at a similar level (1.2195–1.2198 g/cm^3^), corresponding to an average increase of about 1% relative to neat PBS. This phenomenon may be associated with the higher intrinsic density of natamycin and/or its ability to form interactions that promote increased local ordering of the polymer matrix. The stabilization of the density values at higher additive contents may indicate that a limiting level of its integration into PBS has been reached or that partial aggregation of the filler phase occurs.

The observed changes in density are consistent with reports available in the literature. In studies concerning biodegradable polymers modified with natural bioactive additives, density variations are generally minor and rarely exceed 1–2 wt.%, which is attributed to the low additive content and the dominant role of the base polymer microstructure. For PBS, PLA, and PBAT systems containing phenolic acids, polyphenols, or lignin, it has been reported that the observed density changes result primarily from modifications in the degree of crystallinity and chain packing rather than from a classical volumetric filling effect [38,39]. In works focused on active packaging materials, it is emphasized that additives exhibiting antibacterial and antifungal activity—particularly those of natural origin—rarely function as conventional structural fillers; therefore, their influence on density is secondary and depends mainly on morphology and dispersion within the polymer matrix [40].

The melt flow rate (MFR) of neat PBS was 6.3 g/10 min, whereas the incorporation of both caffeic acid and Natamax^®^ resulted in a pronounced increase in MFR, indicating a reduction in melt viscosity under processing conditions. The MFR values of the investigated materials are summarized in Table 3.

The strongest effect was observed for the samples modified with caffeic acid. Already at 1 wt.% of the additive, the MFR increased to 17.3 g/10 min (approximately a 2.7-fold increase relative to neat PBS), while at 3 and 5 wt.% CA it reached 32.2 and 35.2 g/10 min, respectively, corresponding to about a 5–5.5-fold increase compared to the reference material. Such a pronounced, concentration-dependent increase in MFR indicates that caffeic acid significantly modifies the rheological properties of PBS, leading to a substantial reduction in melt flow resistance. This may be associated with a partial decrease in molecular weight (e.g., through degradation reactions initiated by the functional groups of the additive) or with an effective “interchain lubrication” effect that reduces the number of entanglements and facilitates the mobility of macromolecular segments in the molten state. It should be emphasized that such a large increase in MFR, while beneficial from the perspective of processability (e.g., in injection molding or extrusion of thin-walled products), may simultaneously be associated with a reduction in mechanical strength and load-bearing capacity in the solid state, which requires further verification in performance testing.

In the case of the composites containing Natamax^®^, a much milder but stable increase in MFR was observed. The values fall within a narrow range of 11.3–11.7 g/10 min for 1–5 wt.% of the additive, corresponding to approximately a 1.8–1.9-fold increase relative to neat PBS. The absence of a pronounced dependence of MFR on Natamax^®^ content suggests that this additive affects rheology primarily through moderate interfacial interactions or local changes in chain mobility, without inducing significant macromolecular degradation. This type of behavior can be interpreted as a favorable compromise, i.e., a reduction in melt viscosity relative to PBS that facilitates processing, while maintaining a relatively high molecular weight and predictable mechanical properties. The comparison of the results therefore indicates that caffeic acid acts as a strongly flow-modifying additive for PBS, whereas Natamax^®^ exerts a milder effect, enabling controlled rheological modification without drastically altering the nature of the melt.

From a comparative perspective, the obtained MFR results are consistent with the prevailing view in the literature regarding the influence of low-molecular-weight phenolic and/or bioactive additives on the rheological behavior of biodegradable polymers. It has been observed that certain antifungal substances may act as plasticizers or initiate degradation of polyester chains under melt-processing conditions [41,42]. In PLA systems modified, among others, with caffeic acid, pronounced changes in rheological behaviour as well as shifts in thermal parameters have been reported, indicating a “plasticizing–modifying” effect that depends on the polymer matrix and on both the method and form of additive incorporation [43]. At the same time, the literature concerning the use of natamycin in biodegradable polymers shows that the mere presence of an antifungal agent can significantly alter processability and end-use properties; however, the direction and magnitude of this effect strongly depend on compatibilization and on the type of carrier employed (e.g., blends, nanoadditives, multicomponent systems). Moreover, reviews devoted to biodegradable active packaging indicate that an increase in MFR is often interpreted as a consequence of phenol–ester interactions, hydrolysis, or catalytic reactions, particularly in the case of aliphatic polyesters such as PBS or PLA [44].

The tensile test results indicate that both caffeic acid and Natamax^®^ significantly modify the deformation mechanism of PBS, with the direction of the changes depending on the type and concentration of the additive. The mechanical parameters of the investigated materials determined in static tensile testing are summarized in Table 4.

Neat PBS exhibited a tensile strength of 18.9 MPa and very high ductility (ε ≈ 1029%), confirming its pronounced plastic character and ability to undergo extensive deformation prior to failure; this is accompanied by a moderate Young’s modulus (E = 184.6 MPa).

The addition of caffeic acid led to a pronounced shift in the balance between strength and ductility. At 1 wt.% caffeic acid, the tensile strength remained essentially unchanged (18.8 MPa), whereas the elongation at break decreased almost twofold (to approximately 587%) and the elastic modulus decreased to 130.8 MPa. This trend suggests a reduced ability of PBS to undergo stable plastic deformation and stretching (lower “drawability”) without a corresponding strengthening effect. Caffeic acid may thus act as a factor disturbing the microstructure or initiating partial chain degradation (consistent with the strong increase in MFR observed for the caffeic acid series), thereby reducing resistance to large deformations without a significant increase in load-bearing capacity.

At higher caffeic acid contents (3 and 5 wt.%), a significant increase in tensile strength was observed (24.2 and 21.8 MPa, respectively), together with a pronounced stiffening of the material (E ≈ 297–301 MPa), accompanied by a further decrease in elongation at break (to approximately 483% for PBS CA3 and 237% for PBS CA5). This indicates a transition from typically ductile behavior toward a distinctly stiffer and less plastic material. This effect is consistent with the DSC interpretation, in which caffeic acid-containing samples exhibited an increase in T_g_ and a slightly higher degree of crystallinity, indicating restricted mobility of the amorphous phase and more efficient stress transfer in the elastic regime. At the same time, the decrease in ε_B_ suggests that despite the increased load-bearing capacity, the sensitivity to microcrack initiation increases and structural continuity is lost at lower strains, which may be associated with the presence of additive domains, interfacial defects, or increased brittleness resulting from the greater stiffening of the system.

A different behavior was observed for the Natamax^®^-modified materials. At 1 wt.% of the additive, a simultaneous increase in tensile strength (22.7 MPa) and modulus (254.3 MPa) was achieved while retaining relatively high ductility (approximately 589%). This indicates a beneficial modification effect: the material becomes stiffer without a drastic loss of plasticity. This result is consistent with the DSC-observed increase in crystallinity (particularly for PBS N3 in the DSC data) and with the moderate increase in MFR (without the drastic changes seen for caffeic acid), suggesting the absence of strong chain degradation and effective structural reinforcement.

However, at higher Natamax^®^ contents (3 and 5 wt.%), a clear deterioration in strength properties was observed (16.6 and 16.2 MPa), together with a drastic decrease in elongation at break (approximately 158 and 145%), while the modulus remained comparable to or lower than that of PBS (175 and 151 MPa). Such a combination of changes is typical of materials in which structural defects and weakened interfacial cohesion dominate: at higher loadings, the additive may form aggregates, initiate local stress concentrations, and facilitate crack propagation, thereby shortening the plastic deformation stage and reducing both strength and deformability. At the same time, the lack of an increase in modulus for PBS N3–PBS N5 suggests that any increase in crystallinity does not translate into macroscopic reinforcement, because the failure mechanism is controlled by premature cracking at heterogeneities.

The tensile strength, elastic modulus, and elongation at break values obtained in this study for PBS materials modified with caffeic acid and Natamax^®^ are consistent with the behavior patterns widely reported in the literature for active biopolymers containing natural bioactive additives. In particular, the increase in tensile strength and elastic modulus observed in the PBS/CA systems, accompanied by a reduction in plastic deformation capability, agrees with reports on biodegradable polyesters modified with phenolic acids and other phenolic compounds. It has been shown that the incorporation of natural phenolic antioxidants into PLA leads to increased stiffness and strength, which has been attributed to strong interfacial interactions and restricted chain mobility in the amorphous phase, occurring at the expense of elongation at break [45]. Similar relationships have also been reported for PBS and PBAT modified with lignin or nanolignin, where phenolic additives acted simultaneously as stiffening agents and potential initiators of material brittleness [46,47]. The literature repeatedly emphasizes that the decrease in elongation at break in active biocomposites results from the formation of local stress concentrators and a reduced contribution of viscoelastic deformation, particularly at higher contents of organic additives with limited compatibility with the polymer matrix [48]. This mechanism also explains the trade-off observed in the present work between improved strength parameters and reduced ductility in series with higher caffeic acid content.

In the case of Natamax^®^, the results obtained show very good agreement with reports on the incorporation of antifungal agents into biodegradable polymer matrices. Studies on natamycin in polymers such as PLA, PCL, or PBAT indicate the existence of a narrow additive concentration range in which mechanical properties can be maintained or even improved while simultaneously imparting bioactive functionality to the material [49]. Exceeding this range usually leads to a deterioration of strength and elongation at break, which is associated with agglomeration of natamycin particles or its carrier and with weakened interfacial adhesion [32].

Similar trends have also been reported for packaging materials containing natural essential oils and antibacterial compounds, where improvements in biological functionality were often achieved at the expense of mechanical performance at higher additive contents [44,50]. In this context, the results presented here confirm that the mechanical properties of biodegradable polymers containing bioactive additives are strongly governed by the chemical nature of the active substance, its concentration, and its dispersion within the polymer matrix, which constitutes one of the key challenges in the design of active packaging materials.

**Figure 1:**
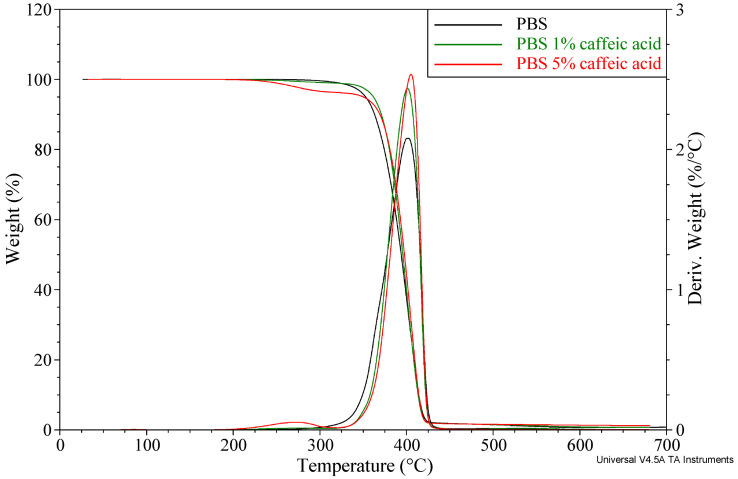
Thermogravimetric curves of samples containing selected contents of caffeic acid.

**Figure 2:**
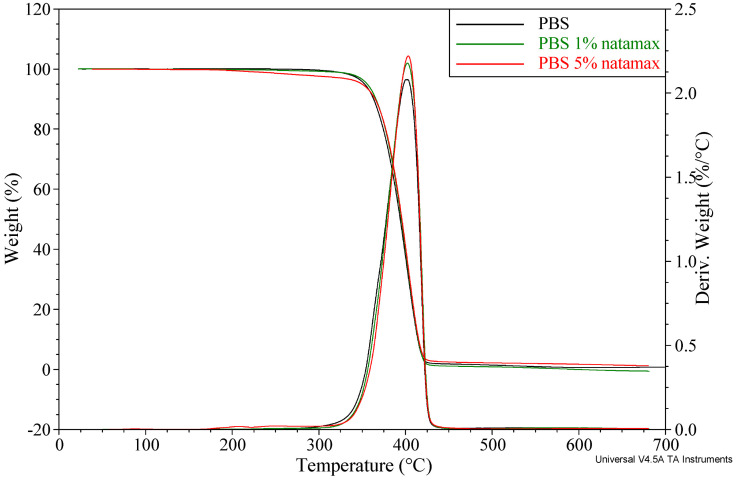
Thermogravimetric curves of samples containing selected contents of Natamax^®^.

Thermogravimetric analysis showed that neat PBS is characterized by high thermal stability, with the onset of major degradation at approximately 352.7 °C (T_5%_), a maximum degradation rate at 402.6 °C, and a low residual mass after combustion (0.8%). The addition of caffeic acid and Natamax^®^ affects the thermal degradation behavior, introducing changes in both the characteristic degradation temperatures and the amount of residue. The TG results for the prepared materials are summarized in Table 5, while representative thermogravimetric curves for selected samples containing caffeic acid or Natamax^®^ are shown in Figure 1 and Figure 2, respectively.

For the materials containing caffeic acid, the T_5_% values show only minor variations and remain within the stability range typical of PBS. The addition of 1 wt.% caffeic acid results in a slight increase in the onset degradation temperature (361.8 °C), whereas at higher contents (3 and 5 wt.%) a decrease is observed, to 355.1 °C and 350.1 °C, respectively. This suggests that a small amount of caffeic acid may exert a stabilizing effect, while at higher loadings the additive lowers this value due to the superposition of the degradation process of caffeic acid itself.

A characteristic feature of the PBS CA3 and PBS CA5 samples is the appearance of an additional degradation step, visible as a T_max1_ signal in the temperature range of 262–273 °C, associated with the thermal degradation of caffeic acid. The intensity of this step (Δm1 = 2.8–3.8%) increases proportionally with additive content, confirming its active participation in the degradation process of the composite. The temperature of the main degradation maximum of PBS (T_max2_ ≈ 401–405 °C) increases with increasing filler content, indicating that caffeic acid slightly improves the thermal resistance of PBS. The slight increase in the residual mass after combustion (1.2–1.3%) relative to neat PBS results from the larger amount of carbonaceous residue formed during the degradation of caffeic acid.

The materials containing Natamax^®^ retained the single-stage degradation behavior typical of neat PBS, with T_max_ in the range of 402–403 °C, indicating that the additive does not significantly interfere with the main degradation mechanism of the polymer. The addition of 1 wt.% Natamax^®^ (PBS N1) does not affect the onset degradation temperature (T_5%_ = 355.8 °C), whereas higher contents (3 and 5 wt.%) lead to a slight decrease to 352.7 °C and 349.5 °C, respectively. The observed decrease may result from the lower thermal stability of Natamax^®^, which influences the shape of the TG curve.

The small mass lost Δ_m1_ (2.2–2.5%) that appear may be associated with the thermal degradation of the additive; however, the absence of a distinct T_max1_ indicates that this process overlaps with the initial stage of PBS degradation. The residue after combustion for samples PBS N1–PBS N5 (0–1.6%) remains at a level comparable to that of neat PBS, confirming that the addition of Natamax^®^ does not introduce a significant amount of thermally stable carbonaceous structures.

The thermal stability of the PBS materials containing caffeic acid and Natamax^®^ obtained in this study are consistent with the general trends reported in the literature on biodegradable polymers modified with natural additives exhibiting antibacterial and antifungal properties. Numerous studies on active biocomposites based on PBS, PLA, and PBAT emphasize that natural additives such as phenolic acids, polyphenols, essential oils, and antifungal agents usually display lower thermal stability than the polyester matrix. As a result, additional mass-loss steps typically appear in the temperature range of 200–300 °C and are attributed to the degradation of the active component [38,51]. Such behavior has been reported, for example, for PBS modified with lignin and its derivatives, where the first degradation stage was associated with the decomposition of phenolic groups, while the main degradation step of PBS remained essentially unchanged.

For phenolic additives, the literature indicates a dual effect on thermal stability. On the one hand, these compounds may initiate earlier mass loss due to their own degradation; on the other hand, owing to their antioxidant properties, they may delay oxidative degradation processes of the polymer matrix at higher temperatures [40]. Similar conclusions have been drawn in review papers addressing the use of natural antioxidants in biodegradable polymers, where it was highlighted that the absence of a significant decrease in the maximum degradation temperature (T_max_) is a key criterion allowing such materials to be processed by melt techniques and applied in packaging applications [41].

In the case of antifungal components such as natamycin, the literature shows that their impact on the thermal stability of biodegradable polymers strongly depends on the mode of incorporation (free molecule, carrier-based systems, or immobilization within the matrix). Studies on active PLA and PCL films containing natamycin have demonstrated that the signal corresponding to its degradation is often weak or partially overlaps with the initial stage of polymer degradation, particularly at low additive contents [50,52]. The authors emphasize that such “masked” degradation of natamycin is advantageous from a technological standpoint, as it does not lead to a significant reduction in processing temperature nor to a sharp decrease in the thermal resistance of the material.

**Figure 3:**
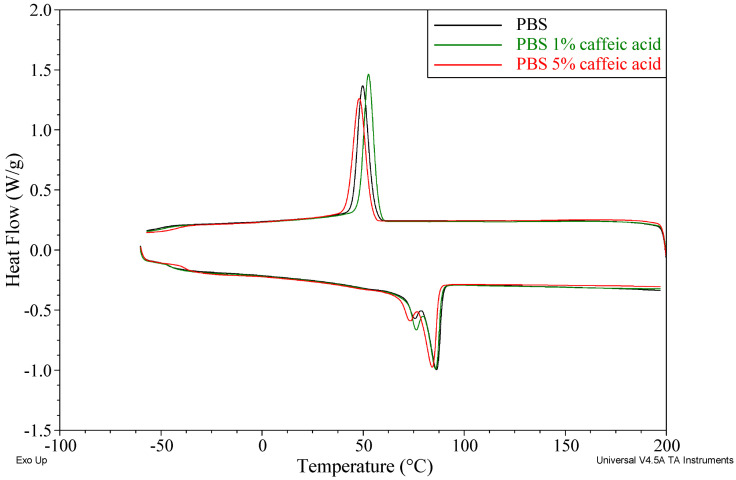
DSC curves of selected samples containing caffeic acid.

**Figure 4:**
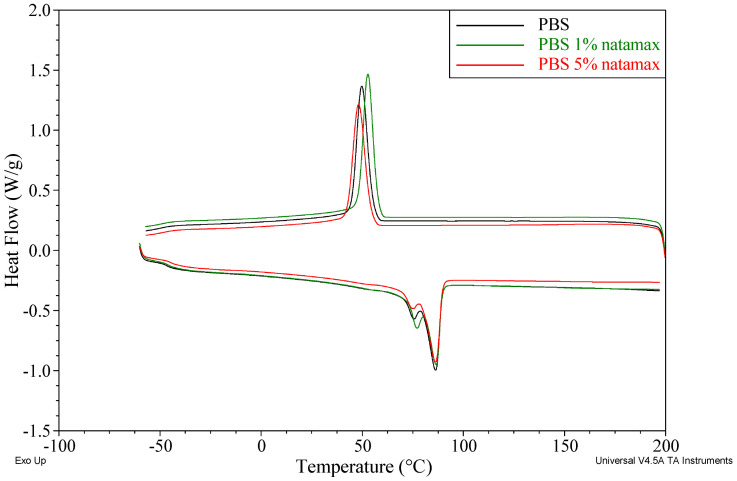
DSC curves of selected samples containing Natamax^®^.

DSC analysis showed that all investigated materials retain the thermal transition behavior typical of PBS, including exothermic crystallization upon cooling (T_c_ ≈ 48–53 °C), a glass transition in the range of approximately −46 to −38 °C, and a two-step melting process with maxima at about 73–77 °C (T_m1_) and 84–87 °C (T_m2_). The incorporation of caffeic acid and Natamax^®^ affects both the positions of these transitions and the degree of crystallinity of the composites. The thermal properties of the prepared materials are summarized in Table 6, while representative DSC curves for selected samples containing caffeic acid or Natamax^®^ are shown in Figure 3 and Figure 4.

Neat PBS is characterized by T_c_ = 49.9 °C, T_g_ = −46.4 °C, a double melting peak with T_m1_ = 75.3 °C and T_m2_ = 86.2 °C, and a melting enthalpy ΔH_m_ = 53.5 J/g, corresponding to a degree of crystallinity X_c_ = 48.5%.

The addition of caffeic acid induces slight shifts in the crystallization and melting temperatures and a pronounced increase in the glass transition temperature. In the PBS CA1 sample, T_c_ increases to 52.6 °C, which may indicate a mild nucleating effect at low additive content, whereas at 3 and 5 wt.% caffeic acid, T_c_ decreases to 50.5 °C and 48.0 °C, respectively, suggesting the coexistence of nucleation effects and restricted chain mobility.

At the same time, T_g_ shifts toward higher values (−45.1 °C for PBS CA1, −39.9 °C for PBS CA3, and −37.9 °C for PBS CA5), indicating a significant reduction in PBS chain mobility in the presence of caffeic acid, most likely due to hydrogen bonding and interfacial interactions. The slight decrease in T_m1_ and T_m2_ with increasing caffeic acid content (T_m1_ down to 73.2 °C and T_m2_ to approximately 84.2 °C for PBS CA5) may reflect the formation of somewhat smaller crystalline lamellae or a higher fraction of crystals with lower perfection.

Despite these changes, ΔH_m_ remains comparable to or slightly higher than that of neat PBS (56–58 J/g), and the degree of crystallinity X_c_ increases to 52.2–53.8%, demonstrating that caffeic acid does not inhibit crystallization and even slightly promotes the crystalline phase content, while simultaneously stiffening the amorphous phase.

For the Natamax^®^ modified materials, similarly small shifts in the crystallization temperature are observed, together with no significant effect on the melting temperature and a more pronounced increase in the degree of crystallinity at selected loadings. For PBS N1, T_c_ is 52.8 °C and T_g_ remains very close to that of neat PBS (−45.7 °C), suggesting that at 1 wt.% Natamax^®^ acts primarily as a mild nucleating agent without substantially affecting macromolecular mobility.

For PBS N3, T_c_ = 50.1 °C accompanied by a marked increase in ΔH_c_ (59.7 J/g) and ΔH_m_ (61.4 J/g), resulting in an increase in X_c_ to 57.4%, the highest value among all investigated samples. This indicates an effective role of Natamax^®^ as a heterogeneous nucleation center promoting the formation of a more developed crystalline structure. At 5 wt.% additive (PBS N5), T_c_ decreases to 48.2 °C and X_c_ remains elevated (54.1%), which may indicate that at higher loadings partial restriction of chain mobility or aggregation of Natamax^®^ reduces the nucleation efficiency. The melting temperatures (T_m1_ ≈ 75.3 °C and T_m2_ ≈ 86.0 °C) remain close to those of PBS, suggesting no significant changes in crystal perfection.

In the case of phenolic additives, numerous studies indicate that the presence of hydroxyl groups promotes strong intermolecular interactions with polyester chains (hydrogen bonding and dipole–dipole interactions), which restricts the mobility of segments in the amorphous phase and manifests as an increase in the glass transition temperature. Such behavior has been repeatedly reported, among others, for PLA and PBS modified with natural phenolic antioxidants, hydroxycinnamic acids, and lignin, where the increase in T_g_ was interpreted as a “stiffening” of the amorphous phase resulting from polymer–additive interactions [38,41]. The increase in T_g_ observed in the present work after the incorporation of caffeic acid fits directly into this framework.

At the same time, the literature emphasizes that phenolic compounds may play a dual role in the crystallization processes of biodegradable polyesters. On the one hand, by limiting chain mobility, they may hinder crystallization; on the other hand, finely dispersed phenolic molecules may act as heterogeneous nucleation sites, leading to an increase in the degree of crystallinity. In studies on PBS modified with lignin and cellulose-based nanoadditives, it was demonstrated that moderate amounts of bio-derived additives promote an increase in PBS crystallinity, whereas higher loadings result in a saturation effect or even a decrease in X_c_ due to deteriorated dispersion and restricted segmental chain mobility [52]. Similar conclusions have been presented in reviews on active packaging materials, which point to the existence of an optimal concentration range of natural additives from the perspective of crystalline nucleation [38].

With respect to Natamax^®^, the maximum degree of crystallinity obtained in this study at an intermediate additive concentration is fully consistent with literature reports on the incorporation of antifungal substances into biodegradable polymers. Studies on active PLA and PCL films, as well as PLA/PBS blends containing natamycin or other antifungal agents, have shown that such additives can act as nucleating agents only within a limited concentration range; once this range is exceeded, agglomeration effects and “dilution” of the crystalline phase dominate, leading to reduced crystallization efficiency [46]. The authors emphasize that such DSC behavior is characteristic of bioactive additives that are not designed as classical nucleating agents but rather as functional components.

Comparison of the DSC results obtained in the present work with literature data confirms that the influence of natural antibacterial and antifungal additives on the thermal properties of biodegradable polyesters is governed by a balance between interactions in the amorphous phase and the effect of heterogeneous nucleation. The observed trends—namely, an increase in T_g_ in the presence of phenolic additives and the existence of a crystallinity maximum at a specific additive concentration—are widely described in the literature and are considered crucial for the design of packaging materials with controlled mechanical and processing properties [38,51].

## 4. Conclusions

In summary, the results clearly indicate that caffeic acid and Natamax^®^ interact with PBS in different ways, leading respectively to a slight decrease or a distinct increase in composite density. This may have important consequences for the subsequent properties of the materials, including their microstructure, crystallinity, and mechanical parameters, and therefore warrants further correlation studies.

The comparison of the MFR results indicates that caffeic acid is a strongly flow-modifying additive for PBS, whereas Natamax^®^ exerts a milder effect, enabling controlled rheological modification without a drastic change in the nature of the melt.

Caffeic acid at 3–5 wt.% acts as a stiffening and reinforcing agent for PBS (increase in σ and E), but at the expense of a substantial loss of deformability, whereas Natamax^®^ exhibits a beneficial effect only at low content (1 wt.%), where it improves strength and stiffness without a drastic decrease in ε_B_. At higher Natamax^®^ contents, the material undergoes a pronounced deterioration of mechanical properties, most likely due to reduced system homogeneity and an increased role of defects that initiate cracking.

Modification of PBS with caffeic acid leads to a two-stage degradation behavior and an increased pyrolytic residue, whereas the addition of Natamax^®^ preserves the single-stage degradation mechanism typical of PBS, with only a slight decrease in the onset degradation temperature at higher additive contents. Both additives induce only minor changes and do not significantly affect the temperature of maximum degradation rate, indicating their limited influence on the main degradation stage of PBS. These results show that both caffeic acid and Natamax^®^ are thermally compatible with PBS, although caffeic acid modifies the degradation pathway to a greater extent by introducing an additional stage associated with its own decomposition.

Both caffeic acid and Natamax^®^ also retain thermal compatibility with PBS without disturbing the fundamental nature of crystallization and melting transitions. Caffeic acid increases T_g_ and slightly increases the degree of crystallinity, which can be associated with stiffening of the amorphous phase while maintaining effective crystallization. Natamax^®^, particularly at 3 wt.%, more strongly increases X_c_, acting as an efficient nucleating agent, with only a minor effect on T_g_ and T_m_. The DSC results, in combination with TGA and density data, indicate different mechanisms of interaction of the two additives with the PBS matrix, which can be exploited in the design of composites with tailored microstructures and performance properties. The present study demonstrates that bio-derived additives such as caffeic acid and natamycin can be successfully incorporated into PBS to create multifunctional biodegradable materials with tailored thermal, mechanical, and processing properties. The results provide quantitative guidelines for selecting additive type and concentration in order to balance activity and material performance, contributing to the rational design of sustainable active packaging systems.

## Figures and Tables

**Table 1:** Designation and composition of the tested samples.

Sample	PBS [wt.%]	Caffeic Acid [wt.%]	Natamax^®^ [wt.%]
PBS	100	-	-
PBS CA1	99	1	-
PBS CA3	97	3	-
PBS CA5	95	5	-
PBS N1	99	-	1
PBS N3	97	-	3
PBS N5	95	-	5

**Table 2:** The density values of the investigated materials.

Sample	ρ [g/cm^3^]
PBS	1.2087 ± 0.0362
PBS CA1	1.2019 ± 0.0340
PBS CA3	1.2032 ± 0.0301
PBS CA5	1.2054 ± 0.0374
PBS N1	1.2217 ± 0.0354
PBS N3	1.2195 ± 0.0183
PBS N5	1.2198 ± 0.0232

**Table 3:** Melt flow rate (MFR) of the investigated materials.

Sample	MFR [g/10 min]
PBS	6.3 ± 0.2
PBS CA1	17.3 ± 0.5
PBS CA3	32.2 ± 0.8
PBS CA5	35.2 ± 1.1
PBS N1	11.3 ± 0.3
PBS N3	11.7 ± 0.2
PBS N5	11.5 ± 0.2

**Table 4:** Mechanical properties determined in static tensile testing.

Sample	σ_M_ [MPa]	ε_M_ [%]	σ_B_ [MPa]	ε_B_ [%]	E [MPa]
PBS	18.9 ± 0.6	1028.7 ± 30.9	18.9 ± 0.7	1028.7 ± 30.9	184.6 ± 5.7
PBS CA1	18.8 ± 0.5	586.8 ± 16.7	18.8 ± 0.5	586.8 ± 16.7	130.8 ± 6.2
PBS CA3	24.2 ± 0.6	482.7 ± 12.2	24.2 ± 0.8	482.7 ± 12.2	296.7 ± 4.5
PBS CA5	21.8 ± 0.7	236.7 ± 7.8	21.8 ± 1.1	236.7 ± 7.8	300.8 ± 9.0
PBS N1	22.7 ± 0.7	589.5 ± 17.9	22.7 ± 0.8	589.5 ± 17.9	254.3 ± 7.4
PBS N3	16.6 ± 0.3	158.2 ± 2.5	16.6 ± 0.3	158.2 ± 2.5	175.0 ± 6.3
PBS N5	16.2 ± 0.3	145.1 ± 3.5	16.2 ± 0.4	145.1 ± 3.5	151.4 ± 7.1

**Table 5:** Thermogravimetric analysis results.

Sample	T_5%_ [°C]	T_50%_ [°C]	T_95%_ [°C]	T_max1_ [°C]	Δm_1_ [%]	T_max2_ [°C]	Δm_2_ [%]	R [%]
PBS	352.7	393.9	418.3	-	-	402.6	99.2	0.8
PBS CA1	361.8	395.8	417.1	-	1.2	401.1	98.0	0.8
PBS CA3	355.1	397.1	416.7	262.2	2.8	404.3	95.8	1.3
PBS CA5	350.1	397.6	417.6	272.6	3.8	405.2	95.0	1.2
PBS N1	355.8	395.2	418.5	-	-	402.1	100.0	0.0
PBS N3	352.7	395.7	419.3	-	2.2	403.2	96.2	1.6
PBS N5	349.5	395.8	419.7	-	2.5	403.1	96.1	1.4

**Table 6:** Thermal properties determined by DSC.

Sample	Cooling		2nd Heating				
	T_c_ [°C]	ΔH_c_ [J/g]	T_g_ [°C]	T_m1_ [°C]	T_m2_ [°C]	ΔH_m_ [J/g]	X_c_ [%]
PBS	49.9	54.6	−46.4	75.3	86.2	53.5	48.5
PBS CA1	52.6	55.8	−45.1	76.1	85.8	57.9	53.0
PBS CA3	50.5	54.6	−39.9	74.2	84.5	55.9	52.2
PBS CA5	48.0	56.0	−37.9	73.2	84.2	56.4	53.8
PBS N1	52.8	53.7	−45.7	77.0	86.5	55.4	50.7
PBS N3	50.1	59.7	−45.9	75.3	86.0	61.4	57.4
PBS N5	48.2	52.8	−44.6	75.3	86.1	56.7	54.1

## Data Availability

The data presented in this study are available upon request from the corresponding author.
